# Causal relationship between human blood omega-3 fatty acids and the risk of epilepsy: A two-sample Mendelian randomization study

**DOI:** 10.3389/fneur.2023.1130439

**Published:** 2023-03-09

**Authors:** Zhen Liang, Yingyue Lou, Zijian Li, Songyan Liu

**Affiliations:** ^1^Department of Neurology, China-Japan Union Hospital, Jilin University, Changchun, China; ^2^Department of Rehabilitation, The Second Hospital of Jilin University, Changchun, China

**Keywords:** omega-3 fatty acids, epilepsy, Mendelian randomization, causal, genetically

## Abstract

**Background:**

Though omega-3 fatty acids reduce seizures in several animal models, considerable controversy exists regarding the association between omega-3 fatty acids and epilepsy in human.

**Objective:**

To assess whether genetically determined human blood omega-3 fatty acids are causally associated with the risk of epilepsy outcomes.

**Methods:**

We conducted a two-sample Mendelian randomization (MR) analysis by applying summary statistics of genome-wide association study datasets of both exposure and outcomes. Single nucleotide polymorphisms significantly associated with blood omega-3 fatty acids levels were selected as instrumental variables to estimate the causal effects on epilepsy. Five MR analysis methods were conducted to analyze the final results. The inverse-variance weighted (IVW) method was used as the primary outcome. The other MR analysis methods (MR-Egger, weighted median, simple mode, and weighted mode) were conducted as the complement to IVW. Sensitivity analyses were also conducted to evaluate heterogeneity and pleiotropy.

**Results:**

Genetically predicted the increase of human blood omega-3 fatty acids levels was associated with a higher risk of epilepsy (OR = 1.160, 95%CI = 1.051–1.279, *P* = 0.003).

**Conclusions:**

This study revealed a causal relationship between blood omega-3 fatty acids and the risk of epilepsy, thus providing novel insights into the development mechanism of epilepsy.

## Introduction

Epilepsy is a common chronic central nervous system (CNS) disorder with substantial morbidity and mortality, which is pathologically characterized by spontaneous, recurrent, and transient CNS dysfunction (1). As estimated, the current prevalence of epilepsy has achieved 1% in the general population, with 80% of people with epilepsy living in low- and middle-income countries, causing a substantial financial burden (2, 3). Aiming to increase the life quality of patients with epilepsy, the goal of all epilepsy treatment is abolishing seizures completely while minimizing the side effects (4).

Nowadays, the most classic treatments for epilepsy are still antiepileptic drugs (AEDs) and surgical intervention (5). Despite the continuous development of AEDs, side effects are still observed and more than 30% of patients with epilepsy progress to refractory epilepsy (5, 6). In these cases, the option is surgical intervention normally by disconnecting rather than removing some brain tissue (7). However, due to the high trauma, high cost, and narrow range of adaptation, there is an urgency to develop new adjuvant treatments, such as dietary therapy (8, 9).

Fatty acids are hydrocarbon chains with a methyl group (–CH_3_) at one end of the molecule and a hydrophilic carboxylic group (–COOH) at the other end. Fatty acids can be divided into polyunsaturated fatty acids (PUFAs), monounsaturated fatty acids (MUFAs), and saturated fatty acids (SFAs), according to the number of carbon-carbon bonds (C=C). PUFAs are fatty acids with multiple double C=C, while MUFAs have one C=C and SFAs have no C=C (10, 11). The two main families of PUFAs include linoleic acid and its derivatives, the omega-6 class, and a-linolenic acid and its derivatives, the omega-3 class. Omega-3 fatty acids mainly consist of eicosapentaenoic acid (EPA) and docosahexaenoic acid (DHA), which are found in fish oils (12). As essential fatty acids, omega-3 fatty acids cannot be synthesized in enough amounts by the body, and therefore they must be supplied by the diet (13). However, there is no consensus on the nutritional requirements of omega-3 fatty acids currently, in large part because their function is not yet fully defined. Previous studies have reported the beneficial effects of omega-3 fatty acids in cardiovascular (14), inflammatory, and autoimmune diseases (15, 16). Meanwhile, omega-3 fatty acids are considered to play antiepileptic roles by stabilizing the neuronal membrane, reducing inflammation, and resisting oxidative damage as the elements of neuronal membrane phospholipids (17–19). In different animal models of epilepsy, omega-3 fatty acids were found to reduce seizures or raise the seizure threshold (20–24). Nevertheless, the roles of omega-3 fatty acids have been inconsistent in several small sample randomized controlled trials (RCTs) (25–30). Given the controversial results and the small sample size of the current RCTs, it is necessary to carry out a larger sample of RCTs or other research strategies with the aim to clarify the relationship between omega-3 fatty acids and human epilepsy.

Mendelian randomization (MR) is a recently developed analytic method, which used genetic variants [single nucleotide polymorphisms (SNPs)] as instrumental variables to mimic the random allocation in the RCT (31, 32). In the case of the absence of reliable RCTs or embarking on new RCTs, MR is an ideal alternative research strategy to clarify the causal relationships between exposures and outcomes (33). In addition, MR can avoid reverse causality as genotype formation precedes disease onset and is unaffected by disease progression (34).

Due to the causal relationships of omega-3 fatty acids on epilepsy being still controversial, we conducted a comprehensive two-sample MR analysis by utilizing genome-wide association study (GWAS) data to clarify the potential causalities in the present study. Findings from this work would not only help to recognize the pathophysiology underlying epilepsy, but also provide reliable evidence for establishing feasible strategies for epilepsy treatment and prevention in clinical practice.

## Materials and methods

### Data acquisition and instruments variables selection

**Figure 1:**
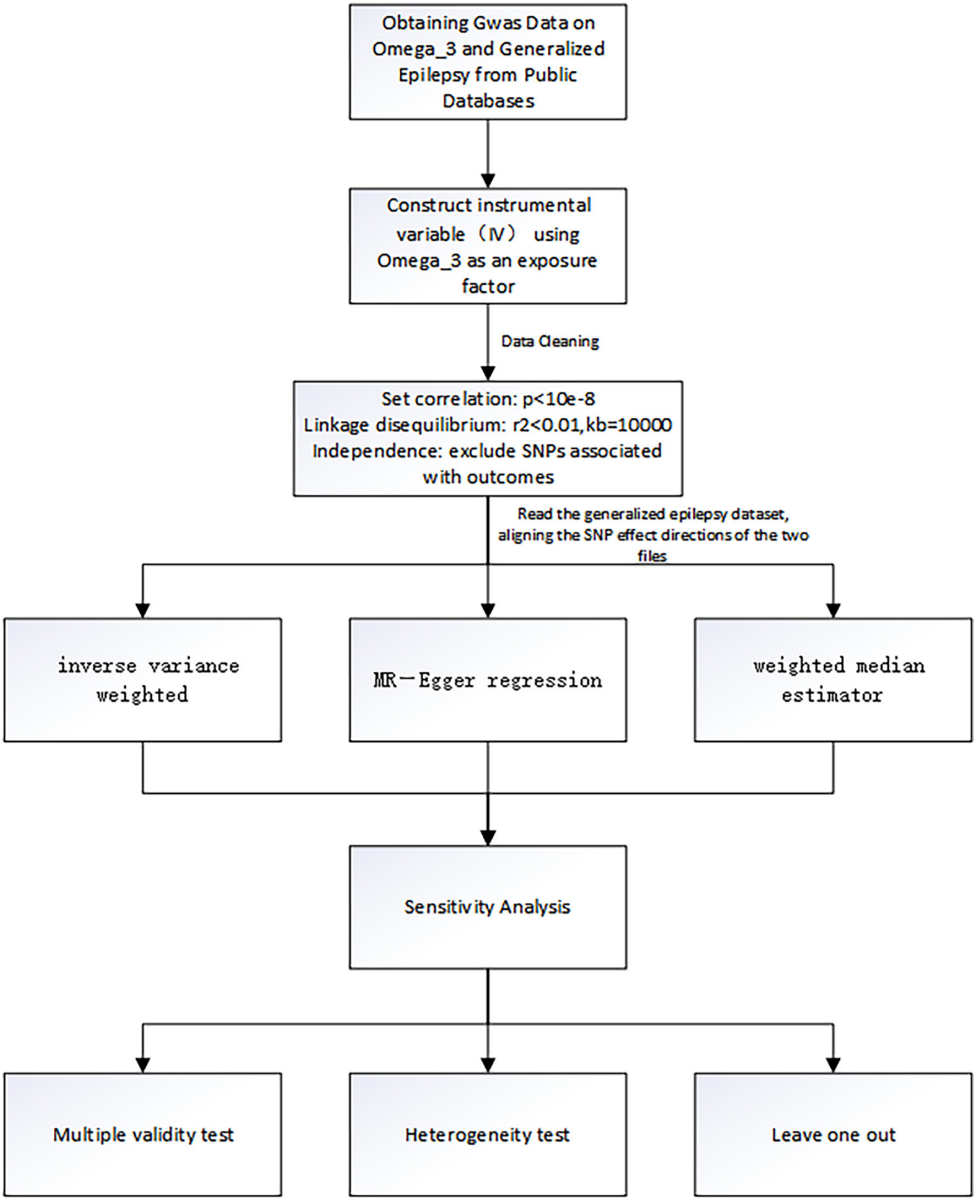
The flowchart of the study. The whole workflow of MR analysis.

The genetic instrumentation for omega-3 was obtained from the public GWAS dataset from the International Federation of Industrial Universities Project (https://gwas.mrcieu.ac.uk), consisting of 114,999 individuals of European ancestry and containing more than twelve million SNPs. The selected SNPs must satisfy the following three criteria: (1) selection of SNPs that were significantly associated with omega-3 fatty acids with a genome-wide significance threshold of *P* < 5 × 10^−8^; (2) removal of interference from linkage disequilibrium, setting kb = 10,000 and *r*^2^ < 0.001, indicating that there is no linkage disequilibrium between SNPs and that the assignment between two SNPs is completely random.; (3) there was no interference from other potential risk factors (35–37). In this study, we used “R studio version 4.1.3” and “MR-PRESSO” packages to test outliers. We set a significance threshold of *P* < 5.0 × 10^−8^ after linkage disequilibrium pruning (*r*^2^ < 0.001 within a 10,000-kilobase window) to obtain the SNPs of omega-3 fatty acids. In addition, we carried out sensitivity analysis to test whether the results of MR analysis were reliable. In the case of *P* > 0.05, it indicated that confounding factors did not affect the outcome, that is, there was no potential bias. Horizontal pleiotropy test was also used to further illustrate this conclusion. The SNPs screening process and the whole workflow of MR analysis are shown in Figure 1.

Genetic datasets for generalized epilepsy (non-focal epilepsy or all types of epilepsy) were obtained from the International League Against Epilepsy Complex Consortium and included all documented cases of generalized epilepsy involving 33,446 people with a total of 3,769 epilepsy patients, containing 480,000 SNPs (38). Summary statistics for the generalized epilepsy dataset are available at the website: https://gwas.mrcieu.ac.uk/datasets/ieu-b-9/.

### Two-sample MR analysis

We systematically assessed the causal relationship between omega-3 fatty acids and the risk of epilepsy using a two-sample MR design. A convincing MR design should comply with three fundamental assumptions: (1) there is a strong association between the IV *Z* and the exposure factor X; (2) the IV *Z* is not associated with any confounders of the exposure-outcome association; (3) the IV *Z* does not affect the outcome *Y* except possibly by association with exposure (39). Among them, the second and third assumptions are collectively known as the independence of horizontal pleiotropy, which could be tested using an array of statistical methods (34).

The inverse variance weighted (IVW), MR-Egger regression, and weighted median (WM) were the main methods used for MR analysis in this study. The traditional IVW method was used as the main MR analysis to assess the causal effect between omega-3 fatty acids and epilepsy. The principle of IVW is to weigh the inverse of the variance of each IV as the weight while ensuring that all IVs are valid, the regression does not consider the intercept term, and the final result is the weighted average of the effect values of all IVs. It is worth noting that IVW can only get correct causal estimates if the SNPs are fully consistent with the three principles of MR studies. Therefore, in the absence of heterogeneity and pleiotropy, we preferentially used the IVW estimates (40). The MR-Egger method differs most from IVW in that the presence of the intercept term is considered when doing regression analysis, and also it uses the inverse of the variance of the outcome as a weight for the fit. We prefer the MR-Egger results when the results are confounded by pleiotropy (41). WM is defined as the median of the weighted empirical density function of the ratio estimates and can output accurate results when more than 50% of the instrumental variables are invalid. That is, we prefer to use the results of the WM method when there is heterogeneity but not pleiotropy (42).

### Horizontal multiplicity and heterogeneity tests

In this study, outliers were detected by using the MR-PRESSO method. If outliers were present, they were removed and the analysis was repeated. The “leave-one-out” sensitivity analysis was performed by removing individual SNPs one at a time to assess whether the variation drove the association between the exposure and outcome variables (43). In addition, to clarify whether there was horizontal pleiotropy in this MR analysis, the MR-Egger intercept test was also performed, and if the intercept term in the MR-Egger intercept analysis was statistically significant, the study was shown to have significant horizontal pleiotropy (41). Finally, this study also used Cochran's *Q* statistic of MR-Egger and IVW for testing the heterogeneity of 24 independent omega-3 fatty acids genetic IVs in the GWAS dataset for generalized epilepsy. Significant heterogeneity in the analysis was demonstrated if Cochran's *Q* statistic test was statistically significant (44). Similar to the meta-analysis, we chose a random effects model to analyze the study process. *P* < 0.05 was considered significant in all studies. All statistical analyses were performed using R studio version 4.1.3, and R packages such as “Two sample MR” and “MR-PRESSO” were used.

### Data availability

All data used in this study were obtained from GWAS summary statistics which were publicly released by genetic consortia. The full period of data collection was from November 1, 2022, to December 1, 2022.

## Results

### Selection of instrumental variables

After a series of quality control steps (Figure 1), 24 independent omega-3 fatty acids SNPs were selected as IVs (*P* < 5.0 × 10^−8^, *r*^2^ < 0.01). Detailed information of selected IVs used in MR analyses is shown in Table 1.

**Table 1:** Detailed information of IVs used in MR analyses.

**SNP**	**Effect allele**	**Other allele**	**Beta**	**Eaf**	***P*val**	**Pos**	**SE**
rs10096633	T	C	−0.0387569	0.123905	1.50E−10	19,830,921	0.00615902
rs10162642	A	G	−0.0488977	0.210115	4.10E−24	58,577,163	0.00501433
rs10184054	G	C	−0.0361462	0.224107	5.60E−15	21,203,877	0.00486548
rs11242109	T	G	0.0240622	0.479016	2.40E−09	131,677,047	0.0040658
rs11563251	T	C	0.0349727	0.110601	3.20E−08	234,679,384	0.00647476
rs117143374	C	T	−0.0370966	0.142254	2.20E−10	40,555,561	0.005847
rs139974673	C	T	0.117987	0.025918	2.30E−21	44,027,885	0.0128075
rs1672811	C	T	0.0251849	0.748488	3.00E−08	15,501,099	0.0046967
rs2072114	G	A	−0.319891	0.122727	1.00E−200	61,605,215	0.00615881
rs2131925	T	G	0.0713932	0.64568	1.20E−66	63,025,942	0.0042536
rs261291	C	T	0.113461	0.356068	1.70E−161	58,680,178	0.00424898
rs35135293	T	C	−0.0208868	0.51675	3.90E−08	20,363,666	0.00408348
rs4000713	A	G	−0.0288196	0.295408	1.00E−11	25,990,597	0.00446039
rs4704834	G	A	0.0289714	0.644056	1.70E−13	156,443,066	0.00424051
rs58542926	T	C	−0.171666	0.074383	1.40E−113	19,379,549	0.00775231
rs6129624	A	G	−0.0257607	0.335237	5.10E−10	39,167,592	0.00437946
rs629301	T	G	0.0382887	0.778033	1.30E−14	109,818,306	0.00488385
rs673335	C	T	−0.0669996	0.159762	1.10E−34	75,450,576	0.00554146
rs7924036	T	G	0.0233527	0.504205	5.50E−10	65,191,645	0.00406452
rs7970695	A	G	−0.0253039	0.620549	1.20E−10	121,423,376	0.00419603
rs964184	C	G	−0.116637	0.867229	8.90E−87	116,648,917	0.00596503
rs9947684	G	A	0.0423222	0.654493	7.40E−25	47,166,694	0.00426979
rs9963974	A	T	0.0307343	0.31897	3.90E−12	47,280,303	0.00435821
rs9987289	G	A	0.0566995	0.909151	3.20E−16	9,183,358	0.00707191

### Causal effects of omega-3 fatty acids on epilepsy

**Figure 2:**
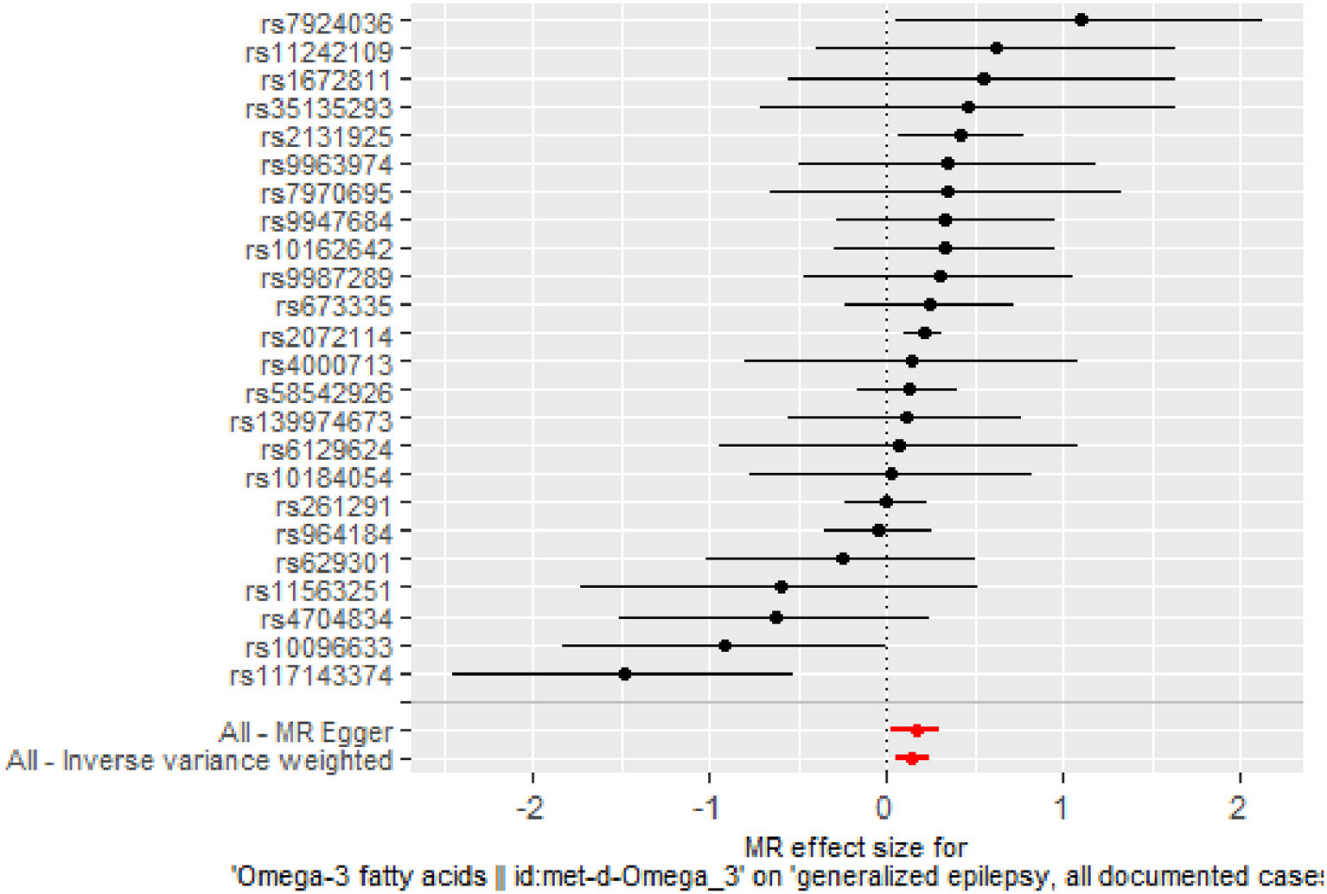
Forest plot of MR analysis of the causal relationship between omega-3 fatty acids and generalized epilepsy. The *x*-axis shows the MR effect size of omega-3 fatty acids on generalized epilepsy. The *y*-axis shows the results of the analysis for each SNP.

**Figure 3:**
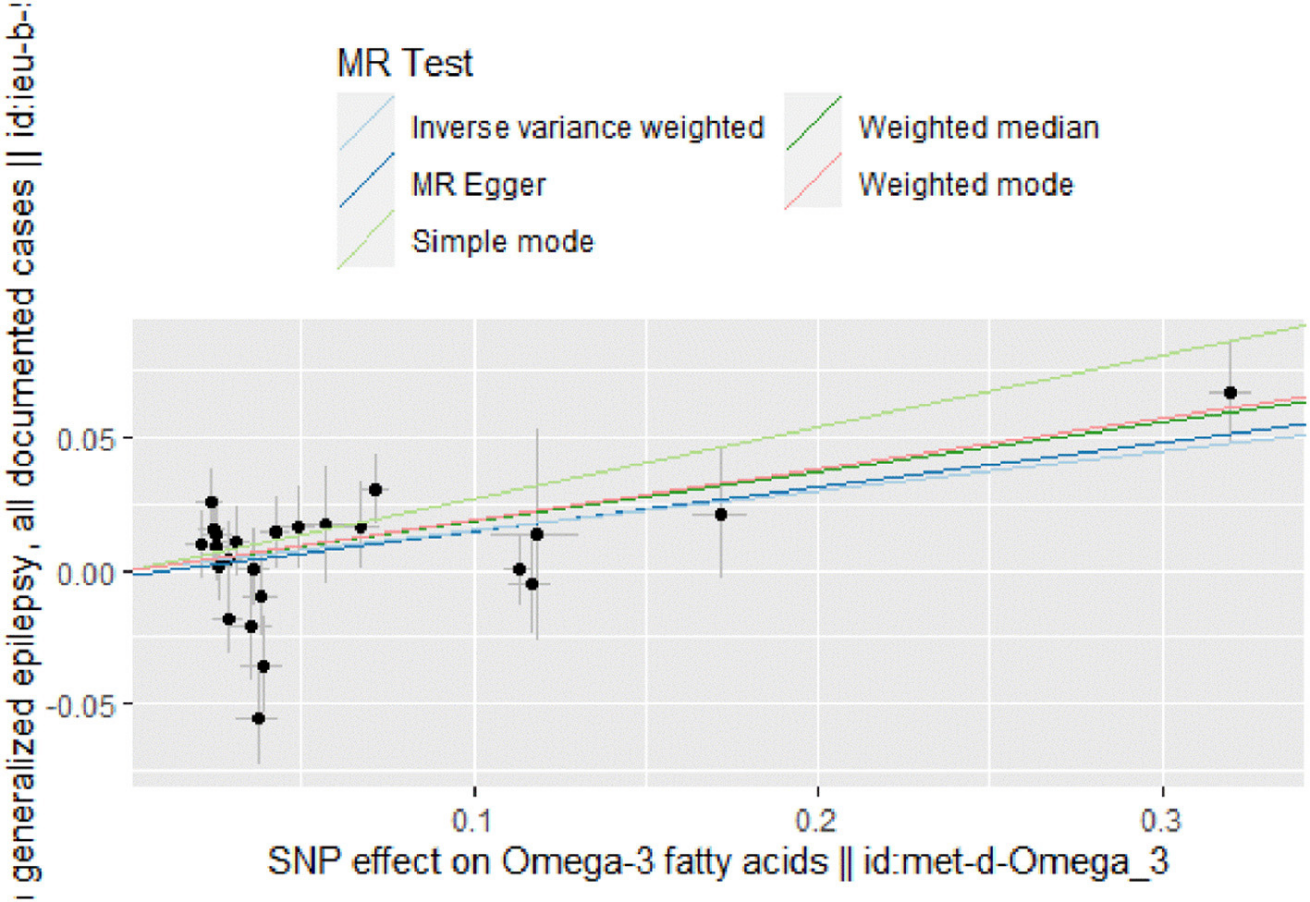
Scatter plot of MR analysis of the causal relationship between omega-3 fatty acids and generalized epilepsy, the beta value represents the slope of the graph; The regression line for MR-Egger, weighted median, IVW, simple mode, and weighted mode is shown.

The IVW results (Figure 2) showed a causal effect association between omega-3 fatty acids and generalized epilepsy [odds ratio (OR) = 1.160, 95% confidence interval (CI) = 1.051–1.279, *P* = 0.003]. Besides, WM (OR = 1.203, 95% CI = 1.079–1.342, *P* = 0.001) and MR-Egger (OR = 1.181, 95% CI = 1.028–1.357, *P* = 0.029) also supported the causal relationship, showing the robustness of the results. Given that all beta values in the results were in the same direction, our analysis suggests that increased levels of omega-3 fatty acids may increase the risk of generalized epilepsy (Figure 3, Table 2).

**Table 2:** MR analysis results between omega-3 fatty acids and generalized epilepsy.

**Method**	**nSNP**	***b***	**SE**	***P*val**	**or**	**or_lci95**	**or_uci95**
MR egger	24	0.166	0.071	0.029	1.181	1.028	1.357
Weighted median	24	0.185	0.056	0.001	1.203	1.079	1.342
Inverse variance weighted	24	0.148	0.050	0.003	1.160	1.051	1.279

### Horizontal pleiotropy and heterogeneity analysis

**Figure 4:**
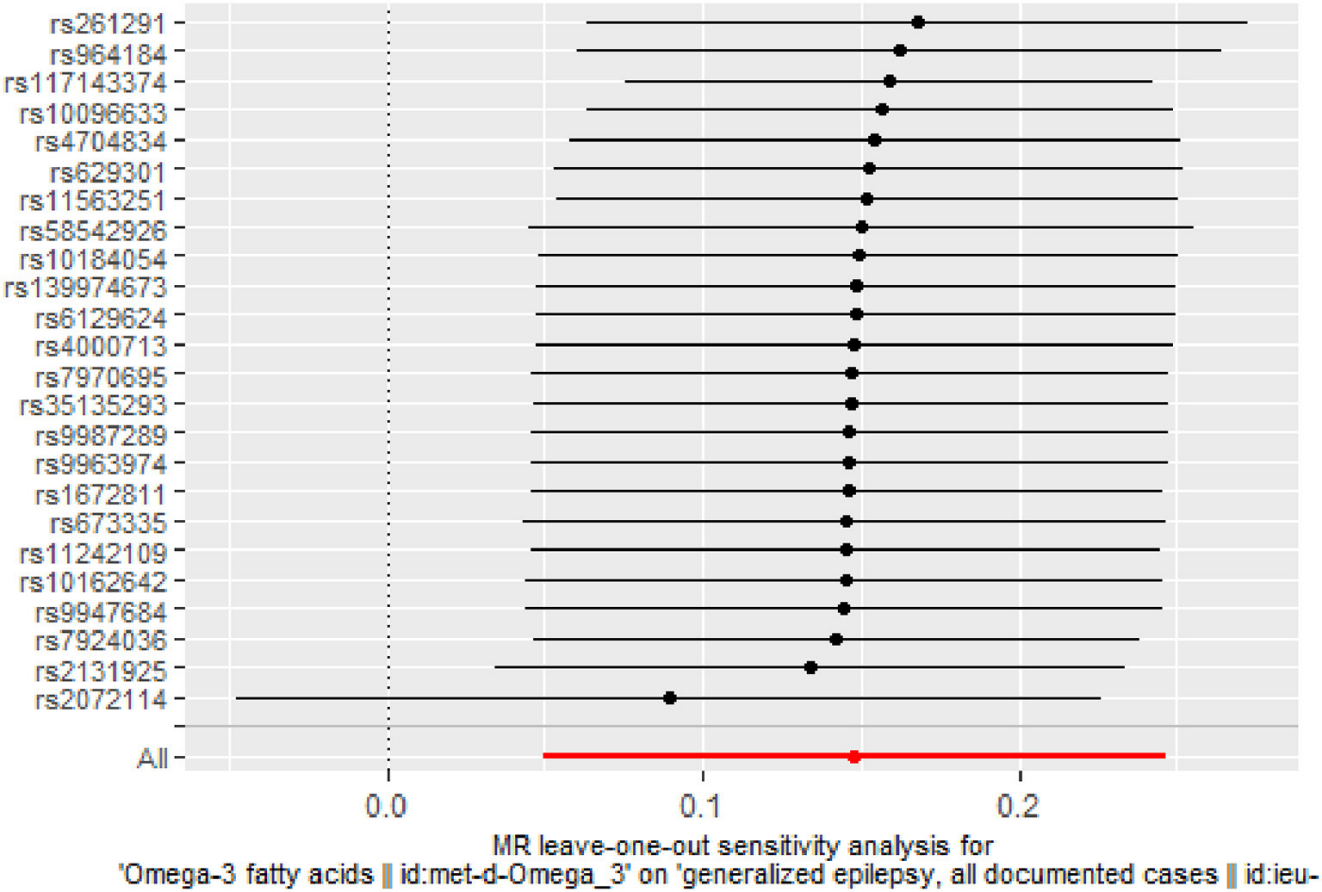
Stability of the causal relationship between omega-3 fatty acids and generalized epilepsy assessed by leave-one-out sensitivity analysis. The x-axis shows the MR leave-one-out sensitivity analysis of omega-3 fatty acids on generalized epilepsy. The *y*-axis shows the analysis of the effect of removing individual SNP on generalized epilepsy.

**Figure 5:**
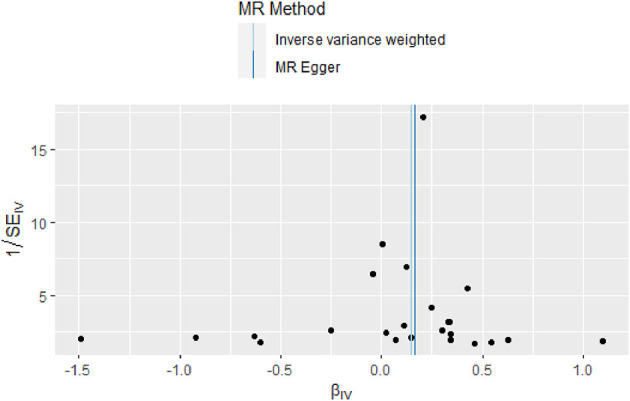
Funnel plot of the causal relationship between omega-3 fatty acids and generalized epilepsy as assessed by MR analysis.

There was no evidence of heterogeneity in IVW analysis (*Q* = 34.579, *P* = 0.042) and MR-Egger regression (*Q* = 34.797, *P* = 0.054). MR-Egger regression showed no evidence of directional pleiotropy across genetic variants (Egger intercept = −0.001; *P* = 0.713). Leave-one-out sensitivity analysis revealed that all black dots were distributed on the right of the dashed line egger intercept = −0.001 (Figure 4). The funnel plot showed that the interpretation of our approach was relatively stable (Figure 5).

## Discussion

To the best of our knowledge, the present study for the first time applied MR analysis to infer the causal relationships between human blood omega-3 fatty acids and the risk of epilepsy in a large population data set of generalized epilepsy. Our results suggested a causal relationship between increased levels of blood omega-3 fatty acids and the risk of epilepsy, which is inconsistent with many previous studies suggesting that omega-3 fatty acids are antiepileptic.

Omega-3 fatty acids are PUFAs with multiple double bonds, mainly including alpha-linolenic (ALA), EPA, and DHA. Most importantly, omega-3 fatty acids are involved in the transmission of nerve impulses and play neuroprotective roles in CNS disorders (45, 46). Specifically, several studies have suggested that omega-3 fatty acids, elements of neuronal membrane phospholipids, may exert antiepileptic effects by stabilizing neuronal membranes, reducing inflammation, and resisting oxidative damage (17–19). These antiepileptic effects have been verified on different animal models of epilepsy (20–24). Unlike the consistency of results from animal experiments, the functions of omega-3 fatty acids in clinical trials remain controversial. Some clinical studies have concluded that fatty acids are beneficial in humans with epilepsy. For example, the ketogenic diet, which we are familiar with, has been proposed to rise the resistance to seizures by increasing omega-3 fatty acids levels, particularly DHA (25, 26). One RCT of different dose of fish oil (EPA and DHA mixture) vs. placebo in 24 participants with drug-resistant epilepsy revealed that low-dose fish oil was associated with a 33.6% reduction in seizure frequency compared with placebo after treatment but high-dose fish oil was no different than placebo in reducing seizures (27). Yuen et al. performed one RCT in which 57 intractable epilepsy patients were randomized to either the omega-3 fatty acids supplement group (30 cases) or the placebo group (27 cases). Results showed that seizure frequency was reduced over the first 6 weeks of treatment in the omega-3 fatty acids supplement group, but this effect was not sustained (28). Not to be overlooked, some RCTs have also found that omega-3 fatty acids do not reduce seizure frequency and even increased seizure frequency compared to placebo, which supports the results of our MR study. In one RCT, researchers concluded that no positive effect of omega-3 fatty acids on seizure frequency was identified (29). What is more, in another RCT, 21 adults with uncontrolled epilepsy were randomized to either a placebo group or the PUFAs supplement group (EPA and DHA mixture). After a 12-week treatment period, seizure frequency increased by 6% in the PUFAs group while decreased 12% in the placebo group (30). The diversity of these RCTs results reveals that the relationship between omega-3 fatty acids and epilepsy is currently unclear. Probably due to the huge difficulty in conducting RCTs, the sample sizes of these studies were small, which also greatly affected the accuracy of the results. In our MR study, relying on the GWAS database, we included a large sample of generalized epilepsy genetic data (*n* case = 3,769), which also gave us a high degree of accuracy in our results. However, we should also note that the current RCTs included patients with mostly refractory or drug-resistant epilepsy, and the GWAS epilepsy genetic data we used was derived from patients with generalized epilepsy, which is not clear what proportion of these are refractory or drug-resistant epilepsy. These subtle differences in the sample may also have an impact on the results. Therefore, future studies targeting the relationship between omega-3 fatty acids and different subtypes of epilepsy are meaningful.

On the other hand, based on the conflicting results of the above RCTs and our MR study, we assume that it might also be necessary to re-examine the traditional view that fatty acids can be antiepileptic or protective of multiple systems in the body. Interestingly, two previous studies also support our assumptions (47, 48). In recent years, the American Diabetes Association has recommended that UK patients with type 2 diabetes mellitus intake oily fish and replace SFAs with PUFAs to prevent diabetes, which appears to be a consensus on the anti-diabetic effects of PUFAs (49). However, in a meta-analysis study, researchers compared the effects of higher intake levels of omega-3 fatty acids, omega-6 fatty acids, and total PUFAs on the incidence of type 2 diabetes mellitus, respectively, and showed no significant reduction in the incidence of type 2 diabetes mellitus. In addition, the investigators also conducted an intervention study in which patients with type 2 diabetes mellitus consuming higher levels of omega-3 fatty acids, omega-6 fatty acids, and total PUFAs. The results also showed no significant reduction in key type 2 diabetes-related indicators (glycated hemoglobin, fasting glucose, fasting insulin, and insulin resistance index) (47). Another meta-analysis measured the association between omega-3 fatty acids and all-cause mortality by three aspects, including mixed prevention, secondary prevention, and patients with the cardio-aid defibrillators. All the results showed that omega-3 fatty acids supplementation could not reduce the risk of all-cause mortality, sudden death, myocardial infarction, cardiac death, or stroke based on relative and absolute measures of association (48). Therefore, it is reasonable to assume that omega-3 fatty acids may not be so magical or beneficial to the organism and that future studies should be designed to be more in-depth and questionable.

The most valuable strengths of this study are its relatively large sample size and the idea of applying a two-sample MR analysis, which minimizes the risk of confounding bias and allows us to take advantage of large-scale epilepsy genetic data. Besides, our study was largely free of reverse causality and residual confounders by using the MR analysis. Specifically, we employ a series of methods to verify any violation of MR assumptions in order to ensure the reliability of MR estimates. The robustness of MR estimates is confirmed by the concordant directions and similar magnitude of various MR models. No evidence of horizontal pleiotropy was found using complementary statistical methods.

It is worth noting that there are still several limitations in this study as well. First, the current GWAS database does not contain the summary-level statistics of epilepsy subcategories, such as drug-resistant epilepsy or refractory epilepsy, making it not possible to further infer the potential relationship between blood omega-3 fatty acids level and risk for subtypes of epilepsy. Second, the data of exposure and outcome in this study were both derived from European databases. Therefore, the results may not be suitable for other ethnic populations. Third, giving that the datasets of exposure and outcome were both derived from European population, there may be a degree of sample overlap. However, as far as we know, there is no good way to evaluate overlapping sample sizes. Finally, although this study suggests that blood omega-3 fatty acids level is causally associated with epilepsy, we should recognize that MR analysis is only a predictive result without verification. Therefore, this causality still needs to be further explored and verified in well-powered RCTs to clarify the existence of causality.

## Conclusion

In conclusion, this MR study indicated that omega-3 fatty acids could increase the risk of generalized epilepsy in a causal way.

## Data availability statement

The original contributions presented in the study are included in the article/supplementary material, further inquiries can be directed to the corresponding author.

## Author contributions

ZLia and SL designed the study. ZLi and YL performed statistical analyses. ZLia wrote the first version of the draft. SL performed visualization and revised the draft. All authors read and approved the final manuscript.

## References

[1] Fisher RS van Emde Boas W Blume W Elger C Genton P Lee P. Epileptic seizures and epilepsy: definitions proposed by the International League Against Epilepsy (ILAE) and the International Bureau for Epilepsy (IBE). Epilepsia. (2005) 46:470–2. 10.1111/j.0013-9580.2005.66104.x 15816939

[2] Banerjee PN Filippi D Allen Hauser W. The descriptive epidemiology of epilepsy-a review. Epilepsy Res. (2009) 85:31–45. 10.1016/j.eplepsyres.2009.03.003 19369037 PMC2696575

[3] Thijs RD Surges R O'Brien TJ Sander JW. Epilepsy in adults. Lancet. (2019) 393:689–701. 10.1016/S0140-6736(18)32596-0 30686584

[4] McNamara JO. Emerging insights into the genesis of epilepsy. Nature. (1999) 399:A15–22. 10392576 10.1038/399a015

[5] Mitchell JW Seri S Cavanna AE. Pharmacotherapeutic and non-pharmacological options for refractory and difficult-to-treat seizures. J Cent Nerv Syst Dis. (2012) 4:105–15. 10.4137/JCNSD.S8315 23650471 PMC3619658

[6] Golyala A Kwan P. Drug development for refractory epilepsy: the past 25 years and beyond. Seizure. (2017) 44:147–56. 10.1016/j.seizure.2016.11.022 28017578

[7] Engel J McDermott MP Wiebe S Langfitt JT Stern JM Dewar S. Early surgical therapy for drug-resistant temporal lobe epilepsy: a randomized trial. JAMA. (2012) 307:922–30. 10.1001/jama.2012.220 22396514 PMC4821633

[8] Kelley SA Hartman AL. Metabolic treatments for intractable epilepsy. Semin Pediatr Neurol. (2011) 18:179–85. 10.1016/j.spen.2011.06.004 22062942

[9] Neal EG Chaffe H Schwartz RH Lawson MS Edwards N Fitzsimmons G. The ketogenic diet for the treatment of childhood epilepsy: a randomised controlled trial. Lancet Neurol. (2008) 7:500–6. 10.1016/S1474-4422(08)70092-9 18456557

[10] Kwan P Brodie MJ. Early identification of refractory epilepsy. N Engl J Med. (2000) 342:314–9. 10.1056/NEJM200002033420503 10660394

[11] Das UN. Biological significance of essential fatty acids. J Assoc Phys India. (2006) 54:309–19. 16944615

[12] Haag M. Essential fatty acids and the brain. Can J Psychiatry. (2003) 48:195–203. 10.1177/070674370304800308 12728744

[13] Tejada S Martorell M Capó X Tur JA Pons A Sureda A. Omega-3 fatty acids in the management of epilepsy. Curr Top Med Chem. (2016) 16:1897–905. 10.2174/1568026616666160204123107 26845549

[14] Connor SL Connor WE. Are fish oils beneficial in the prevention and treatment of coronary artery disease? Am J Clin Nutr. (1997) 66:1020s−31s. 9322583 10.1093/ajcn/66.4.1020S

[15] Wu D Meydani SN. *n* – 3 polyunsaturated fatty acids and immune function. Proc Nutr Soc. (1998) 57:503–9. 10096109 10.1079/pns19980074

[16] Harbige LS. Dietary *n* – 6 and *n* – 3 fatty acids in immunity and autoimmune disease. Proc Nutr Soc. (1998) 57:555–62. 10096116 10.1079/pns19980081

[17] Calder PC. Polyunsaturated fatty acids and inflammation. Prostaglandins Leukot Essent Fatty Acids. (2006) 75:197–202. 10.1016/j.plefa.2006.05.012 16828270

[18] Lauritzen I Blondeau N Heurteaux C Widmann C Romey G Lazdunski M. Polyunsaturated fatty acids are potent neuroprotectors. EMBO J. (2000) 19:1784–93. 10.1093/emboj/19.8.1784 10775263 PMC302016

[19] Yehuda S Rabinovitz S Mostofsky DI. Essential fatty acids and the brain: from infancy to aging. Neurobiol Aging. (2005) 26:98–102. 10.1016/j.neurobiolaging.2005.09.013 16226347

[20] Voskuyl RA Vreugdenhil M Kang JX Leaf A. Anticonvulsant effect of polyunsaturated fatty acids in rats, using the cortical stimulation model. Eur J Pharmacol. (1998) 341:145–52. 9543232 10.1016/s0014-2999(97)01467-2

[21] Yehuda S Carasso RL Mostofsky DI. Essential fatty acid preparation (SR-3) raises the seizure threshold in rats. Eur J Pharmacol. (1994) 254:193–8. 7911428 10.1016/0014-2999(94)90387-5

[22] Musto AE Gjorstrup P Bazan NG. The omega-3 fatty acid-derived neuroprotectin D1 limits hippocampal hyperexcitability and seizure susceptibility in kindling epileptogenesis. Epilepsia. (2011) 52:1601–8. 10.1111/j.1528-1167.2011.03081.x 21569016

[23] Liu SH Chang CD Chen PH Su JR Chen CC Chaung HC. Docosahexaenoic acid and phosphatidylserine supplementations improve antioxidant activities and cognitive functions of the developing brain on pentylenetetrazol-induced seizure model. Brain Res. (2012) 1451:19–26. 10.1016/j.brainres.2012.02.060 22440676

[24] Sierra S Alfaro JM Sánchez S Burgos JS. Administration of docosahexaenoic acid before birth and until aging decreases kainate-induced seizures in adult zebrafish. Brain Res Bull. (2012) 88:467–70. 10.1016/j.brainresbull.2012.04.007 22542883

[25] Bough KJ Rho JM. Anticonvulsant mechanisms of the ketogenic diet. Epilepsia. (2007) 48:43–58. 10.1111/j.1528-1167.2007.00915.x 17241207

[26] Cunnane SC Musa K Ryan MA Whiting S Fraser DD. Potential role of polyunsaturates in seizure protection achieved with the ketogenic diet. Prostaglandins Leukot Essent Fatty Acids. (2002) 67:131–5. 10.1054/plef.2002.0409 12324231

[27] DeGiorgio CM Miller PR Harper R Gornbein J Schrader L Soss J. Fish oil (n-3 fatty acids) in drug resistant epilepsy: a randomised placebo-controlled crossover study. J Neurol Neurosurg Psychiatry. (2015) 86:65–70. 10.1136/jnnp-2014-307749 25201887

[28] Yuen AW Sander JW Fluegel D Patsalos PN Bell GS Johnson T. Omega-3 fatty acid supplementation in patients with chronic epilepsy: a randomized trial. Epilepsy Behav. (2005) 7:253–8. 10.1016/j.yebeh.2005.04.014 16006194

[29] DeGiorgio CM Miller P Meymandi S Gornbein JA. *n* – 3 fatty acids (fish oil) for epilepsy, cardiac risk factors, and risk of SUDEP: clues from a pilot, double-blind, exploratory study. Epilepsy Behav. (2008) 13:681–4. 10.1016/j.yebeh.2008.08.001 18721899

[30] Bromfield E Dworetzky B Hurwitz S Eluri Z Lane L Replansky S. A randomized trial of polyunsaturated fatty acids for refractory epilepsy. Epilepsy Behav. (2008) 12:187–90. 10.1016/j.yebeh.2007.09.011 18086463

[31] Smith GD Ebrahim S. 'Mendelian randomization': can genetic epidemiology contribute to understanding environmental determinants of disease? Int J Epidemiol. (2003) 32:1–22. 10.1093/ije/dyg070 12689998

[32] Burgess S Butterworth A Thompson SG. Mendelian randomization analysis with multiple genetic variants using summarized data. Genet Epidemiol. (2013) 37:658–65. 10.1002/gepi.21758 24114802 PMC4377079

[33] Zuccolo L Holmes MV. Commentary: Mendelian randomization-inspired causal inference in the absence of genetic data. Int J Epidemiol. (2017) 46:962–5. 10.1093/ije/dyw327 28025256

[34] Emdin CA Khera AV Kathiresan S. Mendelian randomization. JAMA. (2017) 318:1925–6. 10.1001/jama.2017.17219 29164242

[35] Yang J Yan B Zhao B Fan Y He X Yang L. Assessing the causal effects of human serum metabolites on 5 major psychiatric disorders. Schizophr Bull. (2020) 46:804–13. 10.1093/schbul/sbz138 31919502 PMC7342080

[36] Cai J He L Wang H Rong X Chen M Shen Q. Genetic liability for prescription opioid use and risk of cardiovascular diseases: a multivariable Mendelian randomization study. Addiction. (2022) 117:1382–91. 10.1111/add.15767 34859517

[37] Choi KW Chen CY Stein MB Klimentidis YC Wang MJ Koenen KC. Assessment of bidirectional relationships between physical activity and depression among adults: a 2-sample Mendelian randomization study. JAMA Psychiatry. (2019) 76:399–408. 10.1001/jamapsychiatry.2018.4175 30673066 PMC6450288

[38] Nature communications. Genome-wide mega-analysis identifies 16 loci and highlights diverse biological mechanisms in the common epilepsies. Nat Commun. (2018) 9:5269. 10.1038/s41467-018-07524-z 30531953 PMC6288131

[39] Boef AG Dekkers OM Le Cessie S. Mendelian randomization studies: a review of the approaches used and the quality of reporting. Int J Epidemiol. (2015) 44:496–511. 10.1093/ije/dyv071 25953784

[40] Pierce BL Burgess S. Efficient design for Mendelian randomization studies: subsample and 2-sample instrumental variable estimators. Am J Epidemiol. (2013) 178:1177–84. 10.1093/aje/kwt084 23863760 PMC3783091

[41] Bowden J Davey Smith G Burgess S. Mendelian randomization with invalid instruments: effect estimation and bias detection through Egger regression. Int J Epidemiol. (2015) 44:512–25. 10.1093/ije/dyv080 26050253 PMC4469799

[42] Bowden J Davey Smith G Haycock PC Burgess S. Consistent estimation in Mendelian randomization with some invalid instruments using a weighted median estimator. Genet Epidemiol. (2016) 40:304–14. 10.1002/gepi.21965 27061298 PMC4849733

[43] Cai J Li X Wu S Tian Y Zhang Y Wei Z. Assessing the causal association between human blood metabolites and the risk of epilepsy. J Transl Med. (2022) 20:437. 10.1186/s12967-022-03648-5 36180952 PMC9524049

[44] Greco MF Minelli C Sheehan NA Thompson JR. Detecting pleiotropy in Mendelian randomisation studies with summary data and a continuous outcome. Stat Med. (2015) 34:2926–40. 10.1002/sim.6522 25950993

[45] Hashimoto M Maekawa M Katakura M Hamazaki K Matsuoka Y. Possibility of polyunsaturated fatty acids for the prevention and treatment of neuropsychiatric illnesses. J Pharmacol Sci. (2014) 124:294–300. 10.1254/jphs.13R14CP 24561447

[46] Kawakita E Hashimoto M Shido O. Docosahexaenoic acid promotes neurogenesis *in vitro* and *in vivo*. Neuroscience. (2006) 139:991–7. 10.1016/j.neuroscience.2006.01.021 16527422

[47] Brown TJ Brainard J Song F Wang X Abdelhamid A Hooper L. Omega-3, omega-6, and total dietary polyunsaturated fat for prevention and treatment of type 2 diabetes mellitus: systematic review and meta-analysis of randomised controlled trials. BMJ. (2019) 366:l4697. 10.1136/bmj.l4697 31434641 PMC6699594

[48] Rizos EC Ntzani EE Bika E Kostapanos MS Elisaf MS. Association between omega-3 fatty acid supplementation and risk of major cardiovascular disease events: a systematic review and meta-analysis. JAMA. (2012) 308:1024–33. 10.1001/2012.jama.11374 22968891

[49] American Diabetes Association. 5. Lifestyle management: standards of medical care in diabetes-2019. Diabetes Care. (2019) 42(Suppl 1):S46–60. 10.2337/dc19-S005 30559231

